# Perceptions towards COVID-19 Vaccines and Willingness to Vaccinate in Nepal

**DOI:** 10.3390/vaccines9121448

**Published:** 2021-12-07

**Authors:** Deepak Subedi, Saurav Pantha, Sanju Subedi, Anil Gautam, Asmita Gaire, Deepak Sapkota, Sachin Sapkota, Milan Kandel, Aabishkar Parajuli, Harishchandra Ghimire, Shristi Ghimire, Janardan Devkota, Santosh Dhakal

**Affiliations:** 1 Paklihawa Campus, Institute of Agriculture and Animal Science (IAAS), Tribhuvan University, Siddharthanagar 32900, Nepal; subedideepu26@gmail.com (D.S.); sauravvet@gmail.com (S.P.); anilg6623@gmail.com (A.G.); asmitagaire1@gmail.com (A.G.); 2 Faculty of Animal Science, Veterinary Science and Fisheries (FAVF), Agriculture and Forestry University, Bharatpur 44200, Nepal; sapkotadeepak2113@gmail.com; 3 School of Public Health, Chitwan Medical College, Chitwan, Bharatpur 44200, Nepal; subedisanju23@gmail.com (S.S.); ghimire.harishchandra@cmc.edu.np (H.G.); 4 School of Medicine, Chitwan Medical College, Chitwan, Bharatpur 44200, Nepal; hinsac12@gmail.com; 5 Himalayan College of Agricultural Sciences and Technology (HICAST), Purbanchal University, Kathmandu 44618, Nepal; milankandel377@gmail.com; 6 Devdaha Medical College, Kathmandu University, Rupandehi 32907, Nepal; aabishkar2000@gmail.com; 7 Johns Hopkins Bloomberg School of Public Health, Baltimore, MD 21205, USA; sghimir2@jhu.edu (S.G.); jdevkot1@jhu.edu (J.D.)

**Keywords:** COVID-19 vaccine, vaccine acceptance, vaccine perception, Nepal

## Abstract

Vaccination is the most effective preventive measure of COVID-19 available at present, but its success depends on the global accessibility of vaccines and the willingness of people to be vaccinated. As the vaccination rollouts are increasing worldwide, it is important to assess public perception and willingness towards vaccination, so that the aim of mass vaccination will be successful. This study aimed to understand public perception towards COVID-19 vaccines and their willingness to get vaccinated in Nepal. This cross-sectional online survey was conducted among 1196 residents of Nepal in August 2021; most of the participants of this online survey were young adults (18–47 years) with university-level education. A total of 64.5% (771/1196) of the participants perceived COVID-19 vaccines to be safe and risk-free, while 68.6% (820/1196) agreed that vaccination would be efficient in the fight against this pandemic. Most of the participants (841/1196, 70.3%) disagreed that people are getting COVID-19 vaccines easily in Nepal, while they agree with the prioritization of older adults and healthcare workers for vaccination. A total of 61.1% (731/1196) of the participants had received at least one dose of the vaccine. Among the unvaccinated, 93.3% (434/465) were willing to get vaccinated when their turn came. The higher confidence of younger adults in vaccines and the vaccination process is encouraging, as that can help educate others who are hesitant or are not positive towards the idea of receiving vaccines. Dissemination of correct and current information, acquisition of enough doses of vaccines, and equitable distribution of vaccines will be required to achieve successful completion of the COVID-19 vaccination campaign in Nepal.

## 1. Introduction

The coronavirus disease 2019 (COVID-19) has imposed cataclysmic changes across the globe. As of late October 2021, there are more than 243 million cases of infections and 4.9 million deaths worldwide [1]. Except for Remdesivir, no other treatment options have been approved against the disease yet, and hence the global community is highly reliant on preventive measures to contain the catastrophe [2]. Vaccines are one of the most effective preventive measures to limit the spread of the disease, and there has been tremendous, accelerated success in the field of COVID-19 vaccine research and development [3]. As of early June 2021, WHO reports 287 vaccine candidates being studied, including 102 already in the clinical trials [4]. Pfizer/BioNtech vaccine Comirnaty (USA), Serum Institute of India (SII)/Covishield (India), AstraZeneca/SK-Bio (AZD1222) (England), Janssen/Ad26.COV 2.S (USA), Moderna COVID-19 vaccine (mRNA 1273) (USA), Sinopharm COVID-19 (China), and Sinovac (Vero Cell) (China) vaccines are already approved by WHO for emergency use [5], and as of 26 October 2021, more than 3.84 billion people have received a dose of a COVID-19 vaccine globally [6]. The success in the development and use of vaccines has provided hope to get rid of the COVID-19 pandemic, but its success is highly dependent on the global availability of vaccines and the willingness of people to accept and receive them [7].

Although vaccines are manufactured and disseminated to minimize the effects of the pandemic, there are studies suggesting people’s hesitancy to vaccine acceptance throughout the globe [8,9,10,11,12]. The success of vaccination campaigns, which aim to achieve herd immunity through mass vaccination, depends largely on the attitude and perception of the public towards available vaccines [10,13,14]. Therefore, it is important to understand people’s perception towards vaccines and willingness to receive them by the related government to formulate proper promotion strategies and lead a successful vaccination campaign [11,15].

After reporting the first COVID-19 case in January 2020, Nepal tried to bring the situation under control with several precautionary measures, including a 5-month-long lockdown during the first wave [16,17]. As of late October 2021, there are 810 thousand cases and 11 thousand deaths in Nepal alongside new variants, vaccination campaigns, and the second wave of the disease [18]. After a year of COVID-19 case diagnosis, in January 2021, Nepal began its first vaccination campaign. Vaccination started with 1 million doses of the Covishield (SII, India) vaccine donated by India [19,20]. Though Nepal needs around 45 million doses of vaccines to cover 72% of its population, the data indicate that, as of late October, just around 15 million doses have been used, with only around 6.7 million people receiving the complete dose and 8.6 million people have settled for a single dose [21].

As vaccines will be available to a larger number of general populations in the future, it is important to assess the perception of the public towards COVID-19 vaccines, vaccine acceptance, and factors associated with them. Therefore, this study aims to determine public perception towards COVID-19 vaccine safety, vaccination campaigns, and vaccine prioritizations; and to understand their willingness to receive COVID-19 vaccines when available.

## 2. Materials and Methods

### 2.1. Ethical Statement

This study was approved by Nepal Health Research Council (NHRC) (Ref. No.: 202). Following the guidelines of the 2013 World Medical Association Declaration of Helsinki Ethical Principles for Medical Research Involving Human Subjects [22], we ensured that participation was voluntary, and informed consent was obtained from the participants, and they could withdraw from the survey at any time.

### 2.2. Questionnaire Design

A structured questionnaire was designed by looking at the scenario of COVID-19 vaccination in Nepal and the study of Seale et al. (2021) [23]. The questionnaire was pre-tested among 20 participants to improve the quality of the questionnaire, and their responses were not included in the study. The questionnaire consisted of demographic information (around 10 questions), questions related to COVID-19 and vaccines (around 10 questions), and questions about COVID-19 vaccine perception (around 9 questions). Four types of vaccines were used in Nepal during the study period: Covishield (SII, India), AstraZeneca (Japan), Verocell (China), and J&J/Janssen (USA), were included in the survey tool. Questions were prepared both in English and Nepali languages.

### 2.3. Study Design, Study Participants, Sample Size, and Sampling

The final pre-tested questionnaire was used to prepare a google survey form and was published online, on 8 August 2021, as a cross-sectional study. Being a Nepalese citizen of age 18 years of above and residing within the country were used as the inclusion criteria for this study. The sample size of the study was determined by a hypothesis of 95% confidence level, with a margin of error of 3% and considering the 29 million population of the country. This gave a computed value of 1067. Further, we added 10% contingency to the sample size; thus, a minimum of 1173 participants were targeted for the study. The online survey was conducted from 8 August to 20 August 2021. We employed a convenient sampling technique, using a web-based survey tool, to reach the target population. The questionnaire was mainly shared via Facebook messenger, Email, and WhatsApp. The online web-based survey was administered both in English and Nepali language so that participants had an option to select the language they preferred.

### 2.4. Data Management and Analysis

Data were collected online, using Google Forms. The final dataset was imported and analyzed by using R studio [24]. Descriptive statistical analysis was performed for sociodemographic characteristics of the study participants and perceptions of the vaccination. Univariate and multivariate logistic regression analyses were conducted to identify factors associated with vaccine perception. Factors assessed included age, gender, education level, employment status, and region of living. All statistical tests were considered significant at a 95% confidence interval with a *p*-value < 0.05. Measures of association were presented as unadjusted and adjusted odds ratios and 95% confidence interval.

## 3. Results and Discussion

A total of 1196 individuals were surveyed in this study. Among them, 644 (53.8%) were males, 874 (73.1%) were between 18 and 27 years of age, 983 (82.2%) had university-level education, 482 (40.3%) were employed, and 658 (55%) were from metro- and sub-metropolitan cities (Table 1). In this study population, 563 (47.1%) participants had taken the COVID-19 test and 205 of them 17.1% of total participants) were tested positive at some point. Nepal started COVID-19 vaccination in January 2021. The vaccine types used in Nepal include COVISHIELD (SII, India), Japanese-made COVID-19 AstraZeneca vaccine, Swedish AstraZeneca, Sinopharm COVID-19 vaccine (Verocell) (China), and J&J/Janssen COVID-19 Vaccine (USA) and as of 26 October 2021, 67,09,905 individuals have received full doses of vaccines [21]. When the survey was carried out, 731 (61.1%) participants had already taken at least one dose of COVID-19 vaccine. Out of 465 participants who were not vaccinated 434, 93.3%, (36.3% of total participants) were willing to get vaccinated when available. Out of 434 participants with the intention of getting vaccinated, 95, 21.9% (7.9% of total participants) were even willing to pay for the vaccine, if available (Table 1).

People’s hesitancy to the use of the vaccines is not a new incident and goes back to the time of smallpox vaccination campaigns [13,14,25]. Vaccine hesitancy: refusal, reluctance, or delay in acceptance of vaccine is considered as one of the ten global health threats [26]. Even in the past, there were low vaccine uptakes against Measles, Mumps, and Rubella (MMR) in European countries due to circulating misinformation that had long-lasting effects in control of the disease [27,28,29]. As far as the rate of vaccine acceptance is concerned, it is highly variable across the globe [30,31]. Earlier studies showed higher (>90%) vaccine acceptance rates in China and Indonesia [32], followed by Brazil (85%), South Africa (80%), Denmark (80%), and South Korea (80%). Mexico, India, and Spain had an acceptance rate of around 75% whereas, in France and Russia, the rates of vaccine acceptance were 60% and 55% respectively [31,33,34]. The United States of America had a difference in the rate of acceptance in different periods with the rate dropping from 74% to 50%, from April 2020 to December 2020 [30,35,36,37]. A study in Qatar showed that around 40% of people were not sure of getting a vaccine [10]. On the contrary, vaccine acceptance rates were reported to be lower in Kuwait (23.6%), the Democratic Republic of Congo (27.7%), and Jordan (28.4%) [38,39]. One earlier study among 266 healthcare workers (HCWs) from a medical college in Nepal reported that only 38.3% of the HCWs were willing to be vaccinated and were concerned about vaccine safety [40]. However, another study showed that Nepal had the highest COVID-19 vaccine acceptance rate among other lower-middle-income countries (LMICs), the average acceptance rate for LMICs being 80.3% [41]. Results in our study were in accordance with the later one and showed that participants in this survey were highly interested in receiving COVID-19 vaccinations.

**Figure 1:**
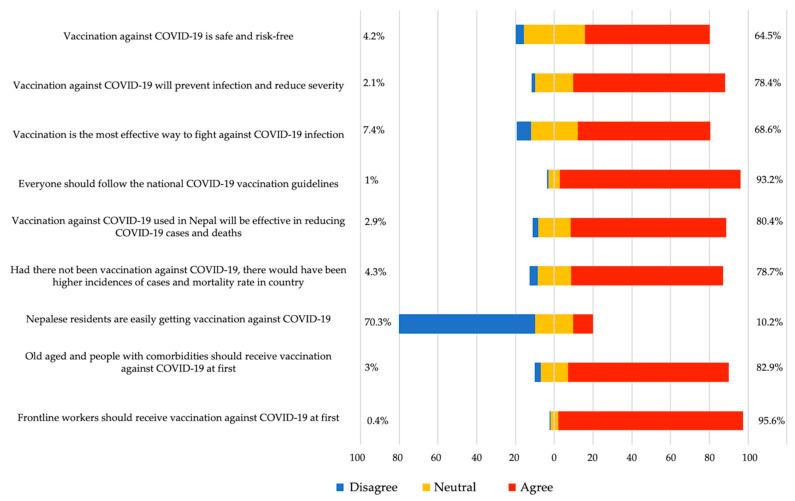
Perception of participants towards COVID-19 vaccines and vaccination in Nepal.

The COVID-19 ‘infodemic’ and circulation of misinformation through various media have raised negative perceptions about COVID-19 vaccines and increased the risk of vaccine hesitancy [42,43,44,45,46]. There are circulating speculations for the imported vaccines and the people doubt the quality standards of the vaccine and fear the side effects [47,48,49]. The public fears the long-term adverse effects of vaccination amid the imbuing rumors [48]. Thus, we wanted to understand the perceptions of survey participants about the COVID-19 vaccine and vaccination in Nepal (Figure 1). Most of the participants (771, 64.5%) perceived COVID-19 vaccines as being safe and risk-free. A total of 938 (78.4%) participants agreed that ‘vaccination against COVID-19 will prevent infection and reduce severity’, while 820 (68.6%) think that ‘vaccination is the most effective way to fight against COVID-19 infection’. To understand their perception of the ongoing COVID-19 vaccination campaign within the country, participants were asked multiple questions (Figure 1). Many of them (1115, 93.2%) agreed that ‘everyone should follow the national COVID-19 vaccination guidelines’. A total of 961 (80.4%) of the participants agreed that vaccination will reduce COVID-19 cases and deaths and 941 (78.7%) perceived that if vaccination was not started, the number of cases and deaths would have been higher in Nepal (Figure 1). There were lots of concerns in the media about the mismanagement of COVID-19 vaccine centers within the country [50]. Concordantly, 841 (70.3%) of the participants in our cohort agreed that people are not getting COVID-19 vaccines easily in Nepal. When asked about their perceptions on prioritization of COVID-19 vaccines, most participants, namely 991 (82.9%) and 1143 (95.6%), respectively, agreed that the older age group and frontline healthcare workers should receive COVID-19 vaccination before others.

COVID-19 vaccines were developed in record time. This raised concerns for many whether any of the processes were bypassed and whether the vaccines are safe [51]. In our cohort, a majority (771, 64.5%) agreed on COVID-19 vaccines being safe and risk-free (Figure 1). To understand if any of the sociodemographic factors were playing role in the participants’ perceptions over safety regarding COVID-19 vaccines, we performed a univariate and multivariate analysis of responses to the question ‘Vaccination against COVID-19 is safe and risk-free’ (Table 2). Age was the only factor that was significantly associated with the perception of COVID-19 safety in both univariate and multivariate analyses. Compared to the reference age group (18–27 years), participants of age 28–37 years (aOR = 1.78, 95% CI: 1.23–2.59, *p* = 0.002) and 38–47 years (aOR = 1.92, 95% CI: 1.08–3.55, *p* = 0.031) reported higher level of agreement on COVID-19 vaccines being safe and risk-free (Table 2). Since the age range 28–47 is likely to have a university education and is the most active age group on the internet, that might have helped the members of this group to gain correct information on COVID vaccines and vaccinations. Other sociodemographic factors, including gender, education, employment status, and geography, were not significantly associated with the perception of COVID-19 vaccine safety (Table 2).

Nepal has an impressive success in childhood immunization in the past indicating that vaccine acceptance at least for children is high [52]. Nepal is acquiring COVID-19 vaccines through the support of countries, such as India, China, Bhutan, Japan, the USA, and the UK, and the Government of Nepal bought 2 million Covishield vaccines (SII, India) from India, paying $4 per dose [53]. On 25 October 2021, Nepal received hundred thousand doses of Pfizer-BioNtech COVID-19 vaccines from the USA via COVAX cost-share scheme of the United Nations [54]. Moreover, Nepal secured 4 million doses of Moderna vaccine through COVAX cost-share scheme and is expected to get it by March 2022 [55]. Since the COVID-19 vaccinations will be feasible to the higher number of the general population in near future, it is important to assess the preferences, perceptions, and acceptance of vaccines to make sure vaccination campaigns will be successful.

In our cohort, 465 (38.9%) participants had not received COVID-19 vaccines yet, and among them, 434 (93.3%) intended to get vaccination when an opportunity arrives, while others did not (Table 1). To understand whether any sociodemographic factors were associated with the intention of getting vaccinated or not, univariate logistic regression analysis was performed for this subset of participants (N = 465). The difference was significant for gender and employment status. Overall, males were less likely (OR = 0.3, CI: 0.12–0.68, *p* = 0.006) to get vaccinated compared to females (Table 3). Contrary to our study, females showed high hesitancy towards being vaccinated in a study in Japan which was associated with greater concerns of vaccine-associated adverse events in females [56]. Such regional differences can be attributed to differences in educational and employment status as well as other sociodemographic factors, exploration of which were beyond the scope of this study. Likewise, unemployed participants had higher (OR = 2.14, CI: 0.93–4.62) will to get COVID-19 vaccines as compared to the employed population (Table 3). In our study, 73.1% of the participants are of age 18–27, and most of them are students. As there is a lesser trend among students to get involved in full or part-time jobs, it is not surprising to see that vaccine acceptance is greater among the unemployed student population, which, in fact, represents young students. The educational status of the participants and geography, however, had no impact on the participants’ willingness to receive the COVID-19 vaccine when available (Table 3).

The strength of our study is the inclusion of a large number of participants compared to earlier studies from Nepal [40,57]. However, these results should be taken with caution. Considering the COVID-19 pandemic protocols, we used a convenient sampling method, and this survey was conducted through online platforms. Though we provided questionnaires both in Nepali and English languages, only those who had internet access and could use it were part of this study. This is the reason for having around 95% of participants aged between 18 and 47 years and over 82% with university-level education. Therefore, this study mostly represents the viewpoint of university-level young adults who have good educational backgrounds and can differentiate between right and wrong through different educational or scientific materials. We have an under-representation of older age groups and people with a lower level of education. However, it is encouraging that the educated young adults in Nepal have a positive perception towards COVID-19 vaccines, as this attitude can be helpful to educate their parents, friends, families, and the hesitant group lacking the confidence to receive COVID-19 vaccination.

## 4. Conclusions

In summary, our study showed that most of the people of age 18 to 85 years in Nepal agreed that COVID-19 vaccines are safe and risk-free and that they will be effective in the fight against this pandemic. Even among the participants who were not vaccinated yet, vaccine acceptance was higher (93.3%). Most of the participants agreed that vaccine priorities should be given to older adults and frontline healthcare workers, as decided by the government, but were concerned that COVID-19 vaccines are not easily available in Nepal, even for the intended group. To achieve successful completion of vaccination campaigns, it will be important for the governmental and other related sectors to make sure correct and current information will be circulated among the public, so that even those who are not interested or unsure about vaccination will be vaccinated. Moreover, COVID-19 vaccines should be made available promptly and should be distributed equitably.

## Figures and Tables

**Table 1:** Sociodemographic characteristics of participants and status of COVID-19 test and vaccination.

Characteristics	Frequency (%)
**Gender**	
Male	644 (53.8)
Female	552 (46.2)
**Age**	
18–27	874 (73.1)
28–37	200 (16.7)
38–47	65 (5.4)
>47	57 (4.8)
**Education level**	
University-level education (bachelor’s, master’s, or PhD)	983 (82.2)
Non university education	213 (17.8)
**Employment status**	
Unemployed	714 (59.7)
Employed	482 (40.3)
Region	
**Cities (metropolitan city or sub-metropolitan city)**	658 (55)
Towns and semi-dense areas (municipality)	406 (34)
Rural areas (rural municipality)	132 (11)
**Previous COVID-19 test**	
No	633 (52.9)
Yes	563 (47.1)
**Having COVID-19 test positive results**	
No	358 (29.9)
Yes	205 (17.1)
**Vaccinated against COVID-19**	
No	465 (38.9)
Yes	731 (61.1)
**Not vaccinated but willing to get vaccinated when available**	
Yes	434 (36.3)
No	31 (2.6)
**Willingness to pay for COVID-19 vaccines**	
No	235 (19.6)
Yes	95 (7.9)
Do not know	104 (8.7)

**Table 2:** Univariate and multivariate logistic regression model of perception on COVID-19 vaccine safety.

Characteristic		Univariate			Multivariable		
	N	OR	95% CI	*p*-Value	aOR	95% CI	*p*-Value
**Age**	1196						
18–27		REF			REF		
28–37		1.6	(1.15–2.25)	0.006	1.78	(1.23–2.59)	0.002
38–47		1.75	(1.01–3.19)	0.053	1.92	(1.08–3.55)	0.031
>47		1.46	(0.83–2.69)	0.2	1.5	(0.82–2.82)	0.2
**Gender**	1196						
Female		REF			REF		
Male		1.06	(0.83–1.34)	0.6	1.08	(0.85–1.37)	0.5
**Educational status**	1196						
Non-university education		REF			REF		
University-level education (bachelor’s, master’s, or PhD)		0.87	(0.63–1.18)	0.4	0.91	(0.65–1.26)	0.6
**Employment status**	1196						
Employed		REF			REF		
Unemployed		0.97	(0.76–1.23)	0.8	1.22	(0.92–1.61)	0.2
**Region**	1196						
Cities (metropolitan city or sub-metropolitan city)		REF			REF		
Rural areas (rural municipality)		0.92	(0.62–1.36)	0.7	0.93	(0.63–1.38)	0.7
Towns and semi-dense areas (municipality)		0.97	(0.75–1.26)	0.8	1	(0.77–1.31)	>0.9

**Table 3:** Univariate logistic regression model of willingness to vaccination among unvaccinated participants.

Characteristic	N	OR	95% CI	*p*-Value
**Gender**	465			
Female		REF		
Male		0.3	(0.12–0.68)	0.006
**Educational status**	465			
Non-university education		REF		
University-level education (Bachelor’s, Master’s, or PhD)		0.69	(0.23–1.70)	0.5
**Employment status**	465			
Employed		REF		
Unemployed		2.14	(0.93–4.62)	0.06
**Region**	465			
Cities (metropolitan city or sub-metropolitan city)		REF		
Rural areas (rural municipality)		0.61	(0.24–1.68)	0.3
Towns and semi-dense areas (municipality)		1.13	(0.49–2.69)	0.8

## Data Availability

Data will be available on reasonable request.
